# Effects of acute ketone monoester ingestion on heart rate, blood pressure and muscle sympathetic nerve activity at rest and stress in healthy adults

**DOI:** 10.14814/phy2.70584

**Published:** 2025-10-05

**Authors:** Erin Seto, Massimo Nardone, Johan S. Thiessen, André L. Teixeira, Julian C. Bommarito, Gaetano C. Pocchi, Devin G. McCarthy, Philip J. Millar

**Affiliations:** ^1^ Human Cardiovascular Physiology Laboratory, Department of Human Health Sciences College of Biological Sciences, University of Guelph Guelph Ontario Canada; ^2^ Human Integrative Physiology of Exercise Laboratory (HIPE‐Lab), Department of Physical Education Federal University of Paraíba João Pessoa Paraíba Brazil

**Keywords:** blood pressure, ketone monoester, muscle sympathetic nerve activity

## Abstract

Exogenous ketone supplementation can impact cardiovascular regulation, but the effects on muscle sympathetic nerve activity (MSNA) remain unknown. In a randomized, crossover, double‐blind design, 19 participants (23 ± 4 years.; female: *n* = 9) ingested an oral ketone monoester (KME; 0.4 g/kg) or isocaloric, isovolumetric placebo. Blood pressure, heart rate, brachial artery blood flow, and MSNA (microneurography; *n* = 14) were assessed at rest before and after ingestion and during two laboratory stressors: 2‐min static handgrip exercise and 2‐min of mental arithmetic. Capillary β‐hydroxybutyrate increased 20 and 40 min post KME ingestion versus placebo (both, *p* < 0.001). Compared to placebo, KME had no effects on resting mean arterial pressure (Placebo: 78.3 ± 9.3 to 80.2 ± 10.5 mmHg; KME: 76.3 ± 8.3 to 79.5 ± 8.8 mmHg; interaction *p* = 0.32), heart rate (Placebo: 64.6 ± 12.3 to 69.6 ± 11.0 bpm; KME: 65.3 ± 10.6 to 72.6 ± 11.3 bpm; interaction *p* = 0.30), or MSNA burst frequency (Placebo: 17.7 ± 7.3 to 17.7 ± 6.7 bursts/minute; KME: 17.5 ± 6.1 to 18.2 ± 7.0 bursts/minute; interaction *p* = 0.72). Resting brachial artery blood flow and vascular conductance changed over time but did not differ between conditions (interactions, both *p* > 0.40). Neuro‐cardiovascular responses during exercise (interactions, all *p* > 0.72) or mental stress (interactions, all *p* > 0.44) did not differ between conditions. Compared to placebo, acute KME ingestion did not modulate neuro‐cardiovascular measures at rest or during stress in healthy young adults.

## 1 INTRODUCTION

The ketone bodies β‐hydroxybutyrate (β‐HB) and acetoacetate are naturally produced during prolonged periods of elevated fat metabolism, that is, starvation or a prolonged low carbohydrate diet (Cahill, 2006). Although ketone bodies are most often recognized as a metabolic fuel (Cahill, 2006), they also possess pleiotropic signaling properties that may alter cardiovascular regulation (Lopaschuk & Dyck, 2023; McCarthy, Chakraborty, et al., 2021). Work in mice has shown β‐HB can act as an antagonist of G‐protein coupled receptors in the sympathetic ganglia, suggesting neural mechanisms may underpin the cardiovascular effects of ketone bodies (Kimura et al., 2011). Ketone body concentration can be acutely elevated through intravenous infusion, with work demonstrating increased heart rate (HR), stroke volume, and cardiac output, along with reduced systemic vascular resistance and possibly blood pressure (BP) in healthy middle‐aged adults and heart failure patients (Gormsen et al., 2017; Nielsen et al., 2019). Alternatively, ingestion of exogenous ketone supplements, for example, ketone salts, diesters, monoesters, represent a practical method to acutely increase blood ketone body concentrations without the need to alter diet or invasive procedures (Stubbs et al., 2017).

A recent meta‐analysis determined that HR and BP responses to acute exogenous ketone ingestion are inconsistent, which may be partially attributable to different study populations, resting versus exercise measurements, and the magnitude of ketosis induced by supplement ingestion (Marcotte‐Chénard et al., 2024). To date, only three studies have examined BP following acute exogenous ketone ingestion in healthy adults, which demonstrated either no change or increased BP after exogenous ketone ingestion (O'Connor et al., 2018; Selvaraj et al., 2022; Oneglia et al., 2023; Wodschow et al., 2023). Notably, in studies that increased blood β‐HB concentration via ingestion of a ketone monoester (KME) supplement, resting HR was increased (Marcotte‐Chénard et al., 2024; McCarthy et al., 2023; Oneglia et al., 2023), as was stroke volume and cardiac output (Oneglia et al., 2023; Selvaraj et al., 2022). Whether changes in cardiac output are a direct result of increased β‐HB or occur secondary to a decrease in peripheral sympathetic outflow caused by β‐HB signaling in the sympathetic ganglia (Kimura et al., 2011) remains unknown. To date, no studies have directly measured muscle sympathetic nerve activity (MSNA) following exogenous ketone ingestion. A recent systematic review concluded that more work is needed to understand the effects of acute exogenous ketone supplementation on HR, BP, and other hemodynamic variables, particularly higher‐quality studies, that is, randomized, crossover, placebo‐controlled trials (Marcotte‐Chénard et al., 2024).

The effects of exogenous ketone supplementation on cardiovascular responses to exercise or other sympathetic stressors have also been understudied. Exogenous ketone supplementation can increase exercising HR during a 30‐min bout of cycling exercise at ventilatory threshold in endurance‐trained adults (McCarthy, Bostad, et al., 2021). In contrast, HR was unchanged during exercise combined with mental stress (i.e., mental arithmetic and a modified Stroop color word test) following acute ketone supplementation (Waldman et al., 2020). Acute exercise is also commonly associated with increases in BP and sympathetic vasoconstrictor outflow and is tested commonly in the laboratory using static handgrip exercise (Dillon et al., 2020). Whether exogenous ketone supplementation can alter these neural or hemodynamic responses to stress has not been investigated.

Therefore, the purpose of this double‐blind, randomized, crossover trial was to study the acute effects of acute KME ingestion on HR, BP, and MSNA at rest and during static handgrip exercise and mental stress. Based on previous experimental (Kimura et al., 2011) and human (Gormsen et al., 2017; Marcotte‐Chénard et al., 2024; McCarthy et al., 2023; Nielsen et al., 2019; Oneglia et al., 2023) work, we hypothesized that acute KME ingestion would increase HR and decrease MSNA at rest and attenuate responses during stress, as compared to placebo.

## 2 METHODS

### 2.1 Participants

**FIGURE 1:**
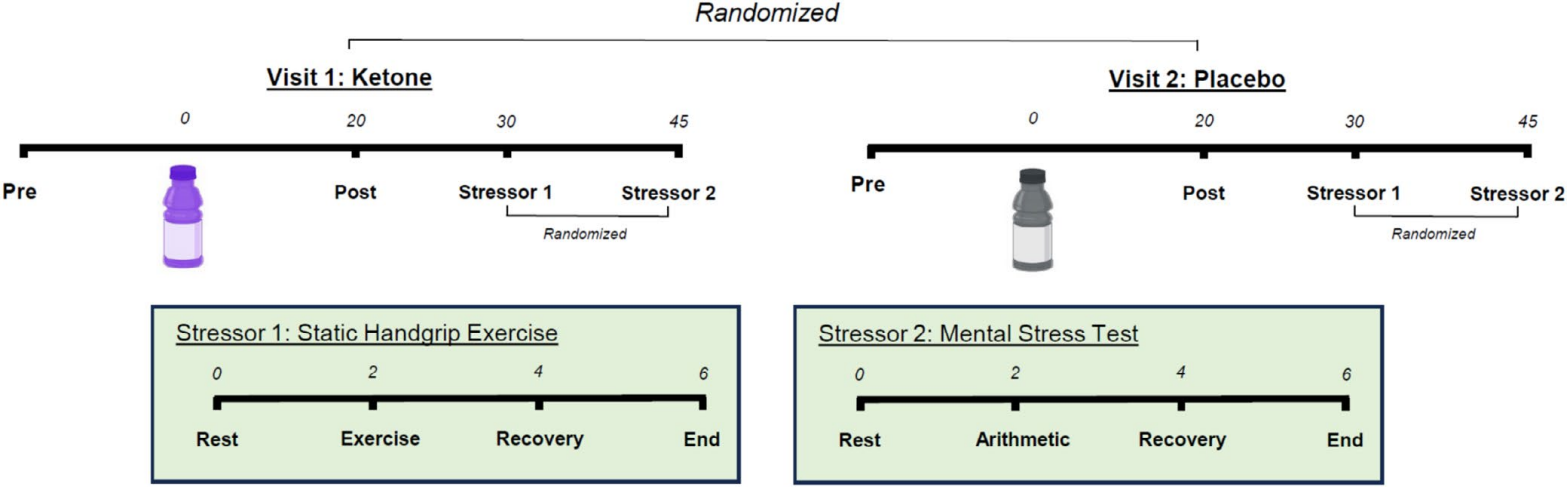
Schematic of study protocol. All time points presented in minutes.

Twenty healthy participants (10 females) between the ages of 18–40 years old were recruited for participation in this study (Figure 1). Exclusion criteria included current or past (within the last 6 months) use of a ketogenic or low‐carb/high‐fat diet, history of any chronic cardiometabolic disease, tobacco smoking, and prescription of pharmacological medications, excluding oral contraceptives. All protocols and procedures were approved by the Research Ethics Board at the University of Guelph (REB #21‐02‐019), and participants provided informed written consent before testing. This study was registered at clinicaltrials.gov (NCT04881526).

### 2.2 Sample size

This study was powered to detect a change in HR and MSNA. Prior work has reported a ∆ + 5 ± 9 bpm increase in resting HR following KME compared to placebo at rest (Nielsen et al., 2019) and a similar magnitude increase in HR (150 ± 11 versus 155 ± 11 bpm [placebo vs. KME]) during 30 min of cycling exercise (McCarthy, Bostad, et al., 2021). Therefore, we anticipated a ∆5 ± 9 bpm increase in HR following KME compared to placebo. Data on the effects of KME on MSNA have not been published, but we powered our study to detect a ∆3 ± 7 bursts/min change in MSNA burst frequency following KME compared to the placebo, with a correlation among repeated measures of *r* = 0.75 (Notay et al., 2017). We estimated the sample size based on using a repeated measures within factor ANOVA with two conditions and two or three measurement time points. Using an anticipated effect size of 0.35 with an alpha = 0.05 and 80% power yielded an estimate of 10–12 participants (G*Power version 3.1). To account for dropouts, missing MSNA data, and to preserve power, *n* = 20 participants were recruited.

### 2.3 Study design and experimental protocol

The present study was a randomized, double‐blinded, placebo‐controlled, crossover design with participants completing two testing visits, separated by a minimum of 30 days. The separation between visits was designed to allow for consistent hormonal phase testing for female participants and for recovery from microneurography. An online random number generator was used to determine visit order (KME or placebo) and the order of the stressors on visit one (mental stress or handgrip exercise). During the second study visit, participants completed the stressors in reverse order from visit one. Participants were instructed to avoid recreational drugs and alcohol (12 h), caffeine (24 h), intense exercise (48 h), and to arrive ≥3 h fasted before each testing visit to limit the influence of exercise and diet on plasma β‐HB levels (Nielsen et al., 2019; Stubbs et al., 2017). Female participants were asked to schedule their testing visits during days 1–5 of their self‐reported early follicular phase or during the placebo phase if taking oral contraceptives (*n* = 4). All testing visits were scheduled ±2 h to limit diurnal variations that may contribute additional variability to cardiovascular measurements.

**FIGURE 2:**
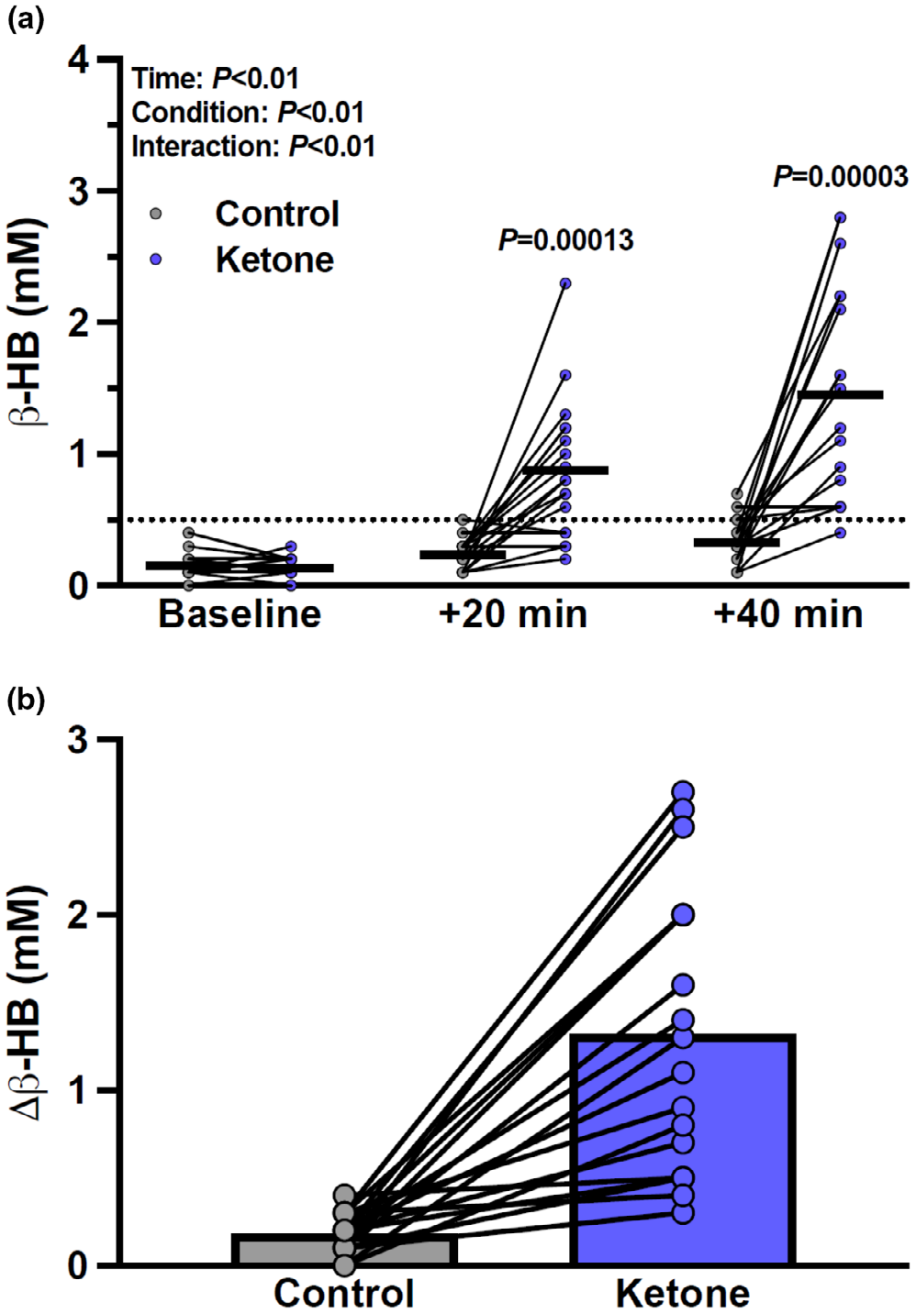
The effect of acute oral ketone monoester (KME) and placebo ingestion on capillary beta‐hydroxybutyrate (β‐HB) concentrations. Panel a: Post measurements occurred 20 and 40 min after consumption, respectively. Panel b: Delta (Δ) changes in β‐HB between the placebo and KME conditions 40 min after ingestion. Values are mean ± SEM. Data analyzed using linear mixed‐effect modeling (*n* = 19).

Upon entering the laboratory, participants were instructed to empty their bladder prior to testing to prevent bladder distention from influencing MSNA and BP (Fagius & Karhuvaara, 1989). Height and weight were subsequently collected. Next, participants were asked to lay supine on a comfortable bed and complete 2–3 brief maximal voluntary contractions, separated by ~2 min, in their left hand. Continuing in a supine position, participants next underwent instrumentation followed by 5 min of rest and a 5 min baseline collection period for HR, BP, MSNA, and brachial artery blood flow measurements. Subsequently, capillary β‐HB was obtained. Participants were then asked to consume the assigned drink and rest for 20 min. This time course was selected to allow for increases in capillary β‐HB concentrations following KME, but minimal changes following the placebo condition, as reported previously (Nielsen et al., 2019; Stubbs et al., 2017, 2019). Post‐measurements were collected, followed by the completion of two acute stressors. Each stress condition consisted of a 2 min baseline, 2 min exercise or mental stress, and a 2 min recovery period. Each stressor was performed in a randomized order and separated by a minimum of 15 min of rest to allow for cardiovascular measures to return to baseline levels. Capillary β‐HB and BP measurements were collected again at the end of the 15 min recovery period. For the exercise stressor, participants completed 2 min of static handgrip exercise at 40% of maximal voluntary contraction, and 2 min of mental arithmetic (serial subtraction), where 13 or 14 was subtracted from a 3‐ or 4‐digit number as the mental stress protocol. A brief overview of the experimental protocol is illustrated in Figure 2.

### 2.4 Measurements

All measurements were collected in the supine posture. Continuous HR was collected with a single‐lead (lead II) electrocardiogram (ADInstruments, Australia). Beat‐to‐beat BP was measured using finger photoplethysmography on the right middle finger (Finometer MIDI, Finapress Medical Systems, The Netherlands), and discrete BP measurements were taken using an automated oscillometric sphygmomanometer from the left brachial artery (BPTru Medical Devices, Coquitlam, BC, Canada). Discrete BP measurement consisted of six measures on a 1‐min cycle, with the average of the last five measurements averaged (BPTru Medical Devices, Coquitlam, BC, Canada). Discrete BP measures were also used to confirm the accuracy of finger BP measurements. Beat‐to‐beat stroke volume was estimated from the BP waveform using the Windkessel model (noninvasive cardiac output extension, ADInstruments, New South Wales, Australia), permitting the calculation of cardiac output. Total vascular conductance was calculated by dividing mean arterial pressure by cardiac output. Capillary β‐HB was measured using finger prick blood samples and a commercially available ketone monitoring device (Precision Libre, Abbott Diabetes Care Ltd., Oxford, UK).

Brachial artery blood flow was measured by duplex ultrasound (Vivid *q*, General Electric, Boston, MA) using a linear‐array probe operating at 13 MHz and 5 MHz for B‐mode and Doppler, respectively. Arterial diameter and blood velocity profiles were recorded and saved using the DVI2USB 3.0 video grabber (Epiphan System, Ottawa, ON, Canada). Arterial diameter and blood velocity were analyzed off‐line with a semiautomated analysis program (Cardiovascular Suite; Quipu, Pisa, Italy). Brachial blood flow was calculated as: blood flow (mL/min) = (MBV) ($\pi r^{2}$) 60; where MBV represents mean blood velocity (cm/s), with *r* representing the radius of the brachial artery (cm). Forearm vascular conductance was calculated as: [brachial artery blood flow/mean arterial pressure].

Microneurographic measures of fibular nerve postganglionic multiunit MSNA were collected as described previously (Notay et al., 2017). A tungsten microelectrode (Frederick Haer, Brunswick, ME) was inserted percutaneously into the fibular nerve posterior to the fibular head, and adjusted until spontaneous, pulse‐synchronous, triangular‐shaped, multiunit bursts of sympathetic activity were distinguishable from the background noise based on a minimum 3:1 signal‐to‐noise ratio. A ground electrode was placed proximally ~3 cm away below the skin surface. Muscle sympathetic activity was confirmed by the absence of responsiveness to unexpected clapping and skin stroking, and reflexive increases in activity in response to an end‐expiration breath hold. The raw MSNA neurogram was amplified, band‐pass filtered (0.7–2.0 kHz), rectified, and integrated to obtain the mean voltage multiunit neurogram (Nerve Traffic Analyzer, Model 662 C‐4; University of Iowa, Iowa City, IA). Visual and audio cues were used to monitor the recording site throughout the duration of the protocol. Sympathetic bursts were identified by an alignment with the time‐shifted cardiac cycle and computed as MSNA burst incidence (bursts/100 heartbeats) and burst frequency (burst/minute). Our laboratory possesses high interday reliability for MSNA measurements, with intraclass correlation coefficients of 0.76 and 0.77 for MSNA burst frequency and incidence, respectively (Notay et al., 2017). Prior work has also established high between‐visit reproducibility of MSNA reactivity during static handgrip exercise (Dillon et al., 2020) and mental stress (Fonkoue & Carter, 2015).

### 2.5 Supplement drink and placebo

Participants ingested 0.4 g/kg of a commercially available KME supplement containing D‐β‐HB and ketone body precursor 1,3‐butanediol (∆G Ketone Performance, TdeltaS, Orlando, Florida). The placebo drink consisted of water, medium‐chain triglyceride oil (NOW Foods Canada, Guelph, Ontario), 1.5 mL digestive bitters (Canadian Bitters, St. Francis Herb Farm, Combermere, Ontario), and 10 dashes of aromatic bitters (House of Angostura, Laventille, Trinidad and Tobago) to match volume, texture, caloric content, and taste of the supplement drink. All drinks were given to participants in an opaque, disposable cup with a lid, consumed using opaque straws. Participants were instructed to consume the assigned drink as quickly as possible and not to react or comment on the taste of the drink. Participants were advised to report any perceived side effects or gastrointestinal discomfort after ingestion.

### 2.6 Data analysis

All continuous data were recorded at a sampling frequency of 1000 Hz, except for the raw MSNA neurogram, sampled at a frequency of 10,000 Hz and stored using LabChart (ADInstruments, New South Wales, Australia). Resting hemodynamic data were calculated as the average over the last 2 min of the baseline. Five‐minute epochs of data from before and after treatments were calculated to determine the effects on resting MSNA, HR variability, and cardiac baroreflex sensitivity. Time–and frequency‐domain measures of HR variability were calculated using LabChart (HRV module, ADInstruments, New South Wales, Australia) and reported as the root mean of successive differences (RMSSD), standard deviation of RR intervals (SDRR), percentage of R‐R intervals that differ by ≥50 ms (pRR50), low‐frequency power (LF, 0.04–0.15 Hz), high‐frequency power (HF, 0.15–0.45 Hz), and the LF/HF ratio. Cardiac baroreflex sensitivity (cBRS) was calculated using the sequences technique (Parati et al., 1988) with a custom R script. The slopes of the up and down sequences did not differ (*p* > 0.10), so only the combined slopes are reported. Neuro‐cardiovascular variables for each stressor were examined by comparing the 2‐min prestressor baseline to the last 30 seconds of the stress period. Brachial artery diameter and mean blood flow velocity were calculated in 1‐sec bins, averaged to derive brachial artery diameter and mean velocity. All data were analyzed blinded to group allocation.

### 2.7 Statistical analysis

To account for potential missing data, all hemodynamic and sympathetic measurements were compared statistically at rest and during each stressor by linear mixed‐effect modeling using the lme4, lmerTest, and emmeans statistical packages in RStudio. Time (pre vs. post or baseline vs. stressor minute 1 vs. stressor minute 2) and condition (placebo and ketone) were included as fixed factors, while participants were included as a random effect to control for within‐participant correlations. When appropriate, post hoc analyses were conducted with a Tukey's adjustment to control for multiple comparisons. Prior work has found that prolonged supine rest can result in small changes in cardiovascular variables and statistically significant main effects of time (Oneglia et al., 2023; Rourke et al., 2025; Teixeira et al., 2023). Thus, the primary statistical comparison of interest was the interaction between time and condition. Placebo‐adjusted correlations were used to test changes in β‐HB versus neuro‐cardiovascular measures at rest. Statistical significance was defined as *p* < 0.05. All data are reported as mean ± SD, unless otherwise stated.

## 3 RESULTS

Twenty participants were recruited for the study between June 2021 and February 2022. One participant withdrew before study initiation. Nineteen participants (23 ± 4 years, 172 ± 8 cm, 75 ± 13 kg, 25 ± 3 kg/m^2^) took part in the study, with two female participants unable to complete their second study visits due to reasons unrelated to the study. Twelve participants were randomized to start with the ketone condition, while mental arithmetic preceded static handgrip exercise in 10 out of 19 participants. Complete microneurographic recordings of MSNA were obtained in 11 (5 females) out of the 19 participants, with three (two females) additional participants having successful recordings from the KME but not placebo visits.

As expected, capillary β‐HB concentrations were higher following KME compared to the placebo at 20 min postingestion (0.9 ± 0.5 vs. 0.2 ± 0.1 mM, *p* = 0.0001) and 40 min postingestion (1.5 ± 0.8 vs. 0.3 ± 0.2 mM, *p* = 0.00003), but not different from placebo at baseline (0.1 ± 0.1 mM and 0.2 ± 0.1 mM, *p* > 0.99) (Figure 2). Capillary β‐HB concentrations following KME differed between both baseline versus 20 min postingestion (*p* < 0.0001) and 20 min postingestion versus 40 min postingestion (*p* < 0.0001) but did not differ between any time points following the placebo (*p* > 0.25). No adverse symptoms were reported.

### 3.1 Resting

**FIGURE 3:**
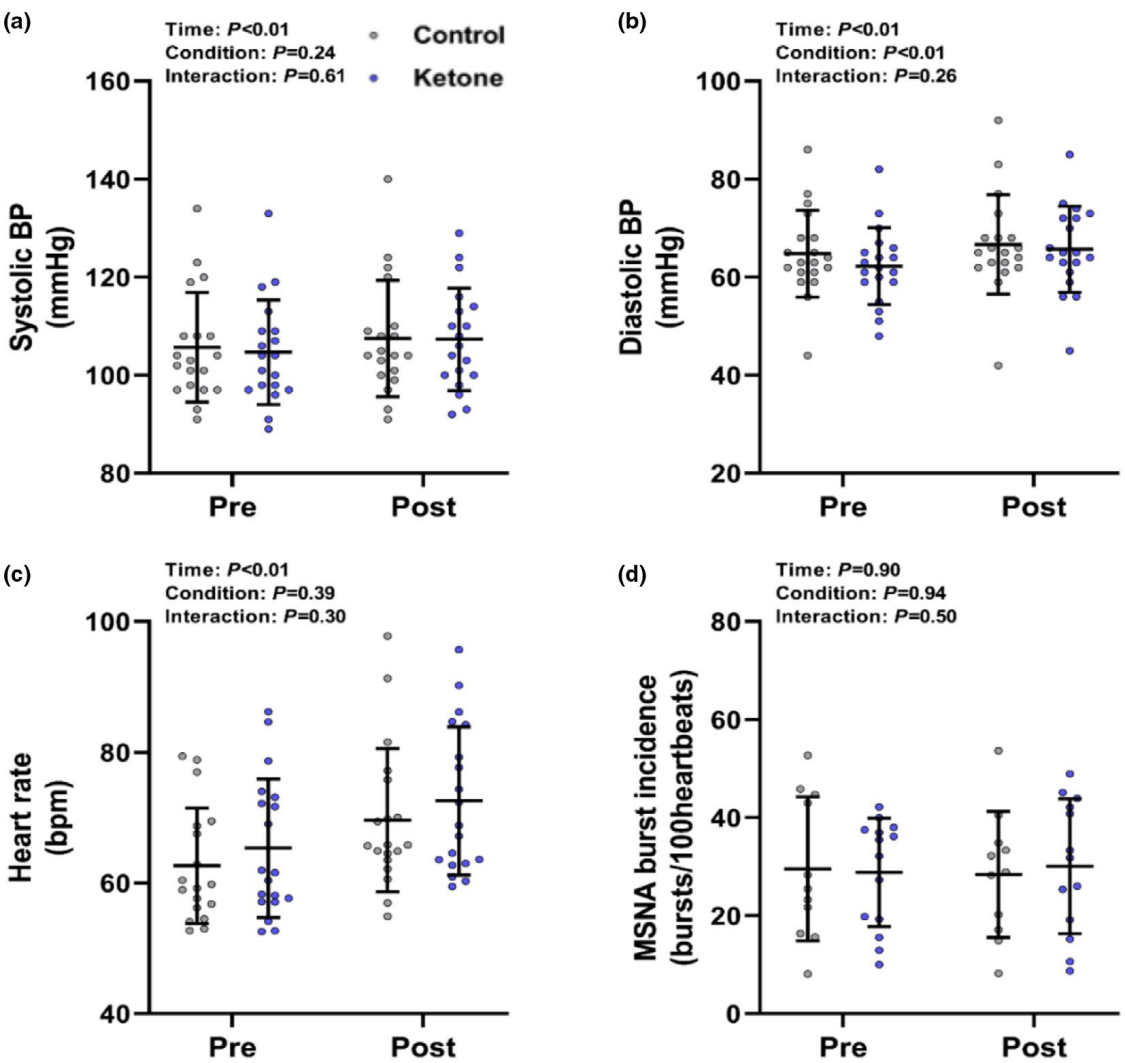
The effects of acute ketone monoester (KME) and placebo ingestion on resting systolic BP (a), diastolic BP (b), HR (c), and MSNA burst incidence (d). Values displayed as individual data points with the lines representing the mean ± SD. Data analyzed using linear mixed‐effect modeling (*n* = 19), with the exception of MSNA data (*n* = 14).

No interaction effects (all *p* > 0.26) were detected following KME or placebo ingestion for resting systolic BP, diastolic BP, HR, or MSNA burst incidence (Figure 3). Similarly, no interaction effects were detected for resting mean arterial pressure, cardiac output, total vascular conductance, brachial artery blood flow, forearm vascular conductance, cBRS, or MSNA burst frequency between ketone and placebo conditions (all *p* > 0.23; Table 1). No interaction effects were detected for indices of HR variability, with the exception of SDRR (*p* = 0.04), though posthoc testing did not reveal any differences between conditions at pre or post time points (both *p* > 0.31). No statistically significant placebo‐adjusted correlations were detected between β‐HB and any neuro‐cardiovascular variable (all *p* > 0.35). It should be noted that several main effects of time were detected, revealing increases in HR, BP, cardiac output, and total vascular conductance, along with reductions in pRR50%, HF power, and cBRS across both conditions (all *p* ≤ 0.01).

**TABLE 1:** Effects of acute oral ketone monoester (KME) or placebo ingestion on resting cardiovascular parameters.

	PLACEBO		KME		*p* values
	Pre	Post	Pre	Post	
MAP, mmHg	78.3 ± 9.3	80.2 ± 10.5	76.3 ± 8.3	79.5 ± 8.8	Interaction = 0.32; Time <0.01; Condition = 0.01
CO, l/min	5.2 ± 1.4	5.8 ± 1.0	5.3 ± 1.3	6.1 ± 1.8	Interaction = 0.63; Time <0.01; Condition = 0.66
TVC, mL/min/mmHg	66.9 ± 16.5	73.4 ± 14.3	69.5 ± 17.0	77.2 ± 24.4	Interaction = 0.82; Time <0.01; Condition = 0.30
Blood flow, mL/min	36.8 ± 24.4	35.9 ± 20.3	43.3 ± 34.8	34.8 ± 19.9	Interaction = 0.39; Time = 0.30; Condition = 0.51
FVC, mL/min/mmHg	0.48 ± 0.4	0.45 ± 0.3	0.57 ± 0.5	0.44 ± 0.2	Interaction = 0.43; Time = 0.17; Condition = 0.48
BF, burst/min	17.7 ± 7.3	17.7 ± 6.7	17.5 ± 6.1	18.2 ± 7.0	Interaction = 0.72; Time = 0.73; Condition = 0.93
RMSSD, ms	82.8 ± 63.5	65.0 ± 45.7	66.6 ± 39.8	63.2 ± 41.2	Interaction = 0.29; Time = 0.12; Condition = 0.55
SDRR, ms	79.5 ± 43.4	74.9 ± 43.4	67.9 ± 27.7	81.1 ± 35.4	Interaction = 0.04; Time = 0.47; Condition = 0.87
pRR50, %	40.9 ± 25.0	31.2 ± 23.0	39.0 ± 28.7	30.5 ± 25.1	Interaction = 0.80; Time <0.01; Condition = 0.97
LF Power, %	28.2 ± 16.4	29.0 ± 17.2	30.8 ± 18.2	25.9 ± 14.4	Interaction = 0.11; Time = 0.48; Condition = 0.80
HF Power, %	39.9 ± 21.4	32.8 ± 20.4	38.9 ± 25.1	27.3 ± 22.2	Interaction = 0.49; Time <0.01; Condition = 0.46
LF/HF	2.2 ± 5.4	1.7 ± 2.5	1.7 ± 2.0	2.9 ± 4.0	Interaction = 0.09; Time = 0.47; Condition = 0.65
cBRS Slope, ms/mmHg	20.5 ± 11.2	16.5 ± 9.2	15.9 ± 7.1	14.6 ± 6.6	Interaction = 0.23; Time = 0.01; Condition = 0.10

*Note*: Mean ± SD.

Abbreviations: BF, MSNA burst frequency; cBRS, cardiac baroreflex sensitivity; CO, cardiac output; FVC, forearm vascular conductance; HF, High‐frequency; LF, Low‐frequency; MAP, mean arterial pressure; pRR50, percentage of R‐R intervals that vary by at least 50 ms; RMSSD, root mean of successive differences; SDRR, standard deviation of RR intervals; TVC, total vascular conductance.

### 3.2 Exercise and mental stress

As expected, mental stress increased BP, HR, cardiac output, brachial blood flow, and vascular conductance (main effect of time, all *p* < 0.01; Table 2). Several main effects for condition were detected, with diastolic BP lower in KME, while HR, cardiac output, total vascular conductance, and MSNA were higher in KME. However, no interaction effects were detected between KME and placebo conditions for the hemodynamic and neural responses to mental stress (all *p* > 0.44; Table 2). Similarly, static handgrip exercise increased BP, HR, cardiac output, brachial blood flow, and vascular conductance, and MSNA (all *p* < 0.01; Table 3). A main effect of condition found lower diastolic BP in the KME condition; however, no interaction effects were detected between KME and placebo conditions for the hemodynamic and neural responses to static handgrip exercise (all *p* > 0.75; Table 3).

**TABLE 2:** Effects of acute oral ketone monoester (KME) or placebo ingestion on hemodynamic and neural responses to mental stress.

	PLACEBO			KME			*p* values
	Baseline	MS1	MS2	Baseline	MS1	MS2	
SBP, mmHg	107.6 ± 11.8	114.1 ± 12.4	112.0 ± 11.4	106.9 ± 9.4	115.2 ± 13.2	114.4 ± 15.3	Interaction = 0.70; Time <0.01; Condition = 0.95
DBP, mmHg	67.3 ± 10.2	71.3 ± 11.0	72.6 ± 10.4	64.3 ± 9.5	70.3 ± 11.4	70.8 ± 12.0	Interaction = 0.82; Time <0.01; Condition = 0.02
MAP, mmHg	80.6 ± 10.5	85.5 ± 10.9	85.6 ± 10.0	78.4 ± 8.9	85.1 ± 11.0	85.2 ± 11.9	Interaction = 0.79; Time <0.01; Condition = 0.13
HR, bpm	70.3 ± 11.8	79.0 ± 12.6	75.1 ± 12.2	75.8 ± 11.1	85.4 ± 14.9	80.0 ± 13.0	Interaction = 0.83; Time <0.01; Condition < 0.01
CO, l/min	5.8 ± 1.0	6.6 ± 1.4	6.1 ± 1.1	6.4 ± 2.0	7.2 ± 2.7	6.5 ± 2.3	Interaction = 0.91; Time <0.01; Condition = 0.04
TVC, mL/min/mmHg	73.2 ± 14.4	78.4 ± 18.2	71.9 ± 14.9	83.7 ± 31.2	87.8 ± 37.0	79.4 ± 34.1	Interaction = 0.92; Time 0.13; Condition < 0.01
Blood flow, mL/min	34.4 ± 17.3	47.3 ± 22.3	39.7 ± 21.9	35.6 ± 21.5	49.7 ± 28.1	41.9 ± 24.1	Interaction = 0.99; Time <0.01; Condition = 0.46
FVC, mL/min/mmHg	0.44 ± 0.26	0.58 ± 0.29	0.50 ± 0.29	0.46 ± 0.26	0.59 ± 0.34	0.51 ± 0.31	Interaction = 0.99; Time = 0.02; Condition = 0.62
BF (burst/min)	16.9 ± 3.9	18.8 ± 7.7	16.1 ± 6.5	20.5 ± 7.0	21.6 ± 10.0	23.6 ± 12.1	Interaction = 0.44; Time = 0.74; Condition < 0.01
BI (burst/100hb)	25.8 ± 8.4	24.6 ± 11.8	22.9 ± 11.7	29.0 ± 11.8	26.1 ± 14.1	29.6 ± 14.0	Interaction = 0.58; Time = 0.71; Condition = 0.055

*Note*: Mean ± SD.

Abbreviations: BF, MSNA burst frequency; BI, MSNA burst incidence; CO, cardiac output; DBP, diastolic blood pressure; FVC, forearm vascular conductance; HR, heart rate; MAP, mean arterial pressure; MS, mental stress minute 1 or 2; SBP, systolic blood pressure; TVC, total vascular conductance.

**TABLE 3:** Effects of acute oral ketone monoester (KME) or placebo ingestion on hemodynamic and neural responses to static handgrip exercise.

	PLACEBO			KME			*p* values
	Baseline	HG1	HG2	Baseline	HG1	HG2	
SBP, mmHg	108.0 ± 11.6	120.7 ± 15.4	138.0 ± 23.0	106.7 ± 10.4	117.3 ± 15.4	134.5 ± 23.9	Interaction = 0.88; Time <0.01; Condition = 0.12
DBP, mmHg	67.0 ± 10.6	77.7 ± 10.8	90.5 ± 12.9	65.2 ± 8.4	74.9 ± 10.8	88.4 ± 15.2	Interaction = 0.93; Time <0.01; Condition = 0.045
MAP, mmHg	80.6 ± 10.7	91.8 ± 11.6	106.2 ± 15.4	79.1 ± 8.7	89.0 ± 10.9	103.7 ± 16.5	Interaction = 0.92; Time <0.01; Condition = 0.06
HR, bpm	69.5 ± 8.8	88.0 ± 11.5	96.9 ± 14.2	72.2 ± 10.3	92.0 ± 12.3	102.2 ± 17.0	Interaction = 0.75; Time <0.01; Condition = 0.11
CO, l/min	5.7 ± 1.1	7.0 ± 1.6	7.7 ± 2.4	5.9 ± 1.6	7.3 ± 2.1	7.7 ± 2.5	Interaction = 0.84; Time <0.01; Condition = 0.70
TVC, mL/min/mmHg	71.6 ± 18.0	77.1 ± 21.9	73.8 ± 26.4	76.2 ± 21.9	82.7 ± 24.8	76.1 ± 26.6	Interaction = 0.85; Time = 0.10; Condition = 0.19
Blood flow, mL/min	35.9 ± 20.3	54.7 ± 43.6	58.5 ± 55.1	34.8 ± 19.9	60.2 ± 58.8	62.1 ± 45.0	Interaction = 0.90; Time <0.01; Condition = 0.66
FVC, mL/min/mmHg	0.45 ± 0.30	0.62 ± 0.53	0.58 ± 0.58	0.44 ± 0.24	0.66 ± 0.63	0.70 ± 0.63	Interaction = 0.76; Time = 0.035; Condition = 0.45
BF (burst/min)	18.8 ± 6.9	24.2 ± 10.2	38.5 ± 19.2	20.7 ± 6.7	27.1 ± 10.0	40.3 ± 18.4	Interaction = 0.98; Time <0.01; Condition = 0.43
BI (burst/100hb)	28.0 ± 12.1	27.9 ± 12.6	40.2 ± 20.8	30.4 ± 10.5	30.2 ± 10.7	40.8 ± 16.8	Interaction = 0.94; Time <0.01; Condition = 0.67

*Note*: Mean ± SD.

Abbreviations: BF, MSNA burst frequency; BI, MSNA burst incidence; CO, cardiac output; DBP, diastolic blood pressure; FVC, forearm vascular conductance; HG, static handgrip exercise minute 1 or 2; HR, heart rate; MAP, mean arterial pressure; SBP, systolic blood pressure; TVC, total vascular conductance.

## 4 DISCUSSION

The present randomized, crossover, double‐blind study investigated the effects of acute ingestion of 0.4 g/kg oral KME on HR, BP, and MSNA at rest and during acute stress in healthy young adults. In contrast to our hypothesis, when compared to the placebo, KME ingestion did not raise HR or lower MSNA and BP at rest. Similarly, we detected no differences in cardiovascular or neural responses during mental stress or static handgrip exercise between the KME and placebo conditions. These findings are in contrast to prior work reporting that acute ketone supplementation can increase heart rate at rest in healthy adults across the lifespan (Myette‐Côté et al., 2019; Nielsen et al., 2019; Oneglia et al., 2023; Rourke et al., 2025) or during exercise in endurance‐training young adults (McCarthy, Bostad, et al., 2021), in addition to reducing systemic vascular resistance (Selvaraj et al., 2022). Collectively, these results indicate that a moderate dose of an exogenous KME increases capillary β‐HB concentrations to ~0.9–1.5 mM and does not alter cardiovascular or neural regulation at rest or in response to mental or physical stress in healthy adults when compared to a placebo supplement.

As a pleiotropic molecule, β‐HB can inhibit histone deacetylase (HDAC) inhibitors and protect against oxidative stress (Shimazu et al., 2013). Ketone bodies, such as β‐HB, have also been identified as regulators of the cardiovascular system (Lopaschuk & Dyck, 2023). The present data adds to a growing body of literature examining how exogenous ketone supplementation may alter resting cardiovascular regulation. To date, the most common observation has been that acute elevations in β‐HB are associated with increases in resting HR (Marcotte‐Chénard et al., 2024; Nielsen et al., 2019; Oneglia et al., 2023; Rourke et al., 2025). In a cohort of young healthy adults, acute ingestion of a large dose of KME (~0.7 g/kg) increased HR, cardiac output, and systolic BP and decreased total peripheral resistance (Selvaraj et al., 2022). The noted reductions in total peripheral resistance could be secondary to a baroreflex‐mediated reduction in sympathetic vasoconstrictor activity caused by the increased systolic BP. In contrast, we found that a moderate dose of KME did not alter resting heart rate, BP, MSNA, estimated cardiac output, heart rate variability, cBRS, or total vascular conductance when compared to the placebo condition. Two key between‐study differences may explain our contrasting results. First, the study by Selvaraj et al. (2022) did not contain a time‐matched control in contrast to our placebo‐controlled double‐blind design. This is important as we detected several main effects of time that could be misinterpreted without a corresponding control condition. Second, in the present study, mean capillary β‐HB measurements increased to ~1.5 mmol/L, and no participants observed a rise greater than 3 mmol/L. A KME dose of 483 mg/kg body mass was shown to increase β‐HB to a mean of 2.1 mmol/L at 45 min postingestion, coincident with increases in HR and cardiac output (Oneglia et al., 2023), whereas the majority of work noting resting cardiovascular effects following acute oral or infusion of β‐HB have raised plasma β‐HB concentrations to ~3–4 mmol/L (Gormsen et al., 2017; Nielsen et al., 2019; Selvaraj et al., 2022). This is important as the cardiovascular effects of β‐HB appear dose‐dependent in patients with heart failure with reduced ejection fraction (Nielsen et al., 2019) and healthy young adults (Rourke et al., 2025). Future work is necessary to determine if larger doses of KME are required to acutely modify resting neural and cardiovascular measures in healthy adults.

To date, the most widely studied application for exogenous ketone supplementation has been as an ergogenic aid (Evans et al., 2022). However, the effects of exogenous ketone supplementation on cardiovascular responses to exercise or other physiological stressors have been less studied. Acute ingestion of 0.3 and 0.6 g/kg body mass KME raised blood β‐HB concentration to ~2–4 mM and increased heart rate during fixed workload exercise compared to a placebo (Bone et al., 2025; McCarthy et al., 2023; McCarthy, Bostad, et al., 2021). In contrast, acute exogenous ketone ingestion that raised blood β‐HB concentration up to ~1.5–2.6 mM did not affect exercise heart rate (Evans & Egan, 2018). Other trials have reported reductions in exercising heart rate, which were likely secondary to accompanied reductions in time‐trial power output (Leckey et al., 2017; McCarthy et al., 2023). The present study found no differences in heart rate during a fixed duration static handgrip contraction at 40% of maximal voluntary contraction when comparing KME to the placebo condition, in addition to no differences in exercise BP or MSNA. Extending these findings, we also found no differences in neural and cardiovascular responses to a serial subtraction mental stress test between the conditions. This aligns with prior work reporting that heart rate during a combined exercise plus mental stress challenge was unchanged (Waldman et al., 2020). Whether the between‐study variability in cardiovascular responses is impacted by factors such as KME dose, sex, exercise mode, or exercise training status warrants further study.

We acknowledge several methodological considerations. First, the placebo beverage was free of exogenous ketone bodies but did contain a small amount of medium‐chain triglycerides, which at high doses can stimulate ketone production (Lin et al., 2021). The mean capillary β‐HB concentration in the placebo group was not higher than ~0.3 mM, which is similar to reports from a placebo‐controlled study using only water and flavoring (Oneglia et al., 2023), and we did not observe any relationships between changes in β‐HB and cardiovascular variables, despite prior work showing a dose‐dependent effect on heart rate (Rourke et al., 2025). β‐HB concentrations were below the 0.5 mM cut‐off for ketosis, and the small increase over time may be reflective of the overnight fast and length of the study visit. We detected several main effects for time, which we cannot exclude were caused by an acute response to the bitter flavor or mild gastrointestinal distress elicited in both conditions (McCarthy, Bostad, et al., 2021; McCarthy, Chakraborty, et al., 2021; Stubbs et al., 2019). Participants did not report any symptoms of gastrointestinal symptoms and our rationale for the placebo supplement was to create a beverage that was isocaloric with a similar bitter taste to the KME. The presence of main effects for time and lack of stability in cardiovascular measures over time in the placebo condition may have led our study to be underpowered to detect differences between conditions over time. The observation that total vascular conductance increased over time without a corresponding change in MSNA suggests a nonneural mechanism of action. We did not measure changes in blood glucose, blood lipids, or fuel oxidation; therefore, we are unable to determine the contribution of β‐HB supplements to whole‐body metabolism. This is important as the interactions between β‐HB and the sympathetic nervous system through GPR41 may vary by species. In humans, GPR41 is found in sympathetic ganglia located in abdominal and thoracic regions and adipose tissue, and thus may contribute more to metabolism and energy homeostasis (Inoue et al., 2014; Kimura et al., 2011). We did not calculate measures of MSNA burst amplitude, owing to the need to make adjustments to the microelectrode position in several participants between recording periods, making it difficult to complete appropriate normalization procedures to account for potential differences in distance to the discharging sympathetic fibers. The present study also investigated oral KME ingestion and may not be translatable to intravenous administration of β‐HB, which has generally demonstrated larger and distinct cardiovascular effects (Gormsen et al., 2017; Nielsen et al., 2019). Lastly, the present study investigated young healthy adults, and the results may not translate to older adults or clinical populations.

## 5 CONCLUSION

Oral ingestion of 0.4 g/kg body mass of a KME supplement increased capillary β‐HB concentrations in young, healthy adults, but did not alter HR, BP, or MSNA at rest or in response to the exercise or mental stress when compared to a placebo containing medium chain triglyceride oil and bitters. Future work is necessary to examine dose‐dependent responses of exogenous ketone ingestion on neuro‐cardiovascular regulation.

## AUTHOR CONTRIBUTIONS

ES and PJM contributed to the conceptualization and design of the study. Data collection was performed by ES, MN, ALT, JCB, PJM. Data analysis was completed by ES, MN, JST, ALT, and GCP. Statistical analysis was performed by ES, MN, JST, and GCP. ES, MN, JST, GCP, DGM, and PJM interpreted the results. The original manuscript draft was written by ES. All authors commented on and revised the manuscript, and all authors approved the final manuscript.

## FUNDING INFORMATION

P. J. Millar receives research support from the Natural Sciences and Engineering Research Council of Canada (NSERC) Discovery Grant program, the Canada Foundation for Innovation, and the Ontario Ministry of Research, Innovation, and Science. P. J. Millar is a recipient of an Early Researcher Award (18‐14‐288) by the Ontario Ministry of Economic Development, Job Creation, and Trade. M. Nardone was supported by a Canadian Institutes of Health Research Frederick Banting and Charles Best Canada Graduate Scholarship.

## CONFLICT OF INTEREST STATEMENT

No conflicts of interest, financial or otherwise, are declared by the authors.

## Data Availability

The data that support the findings of this study are available from the corresponding author upon reasonable request.
